# Academy of Medicine and Surgery, Richmond, Va.

**Published:** 1894-02

**Authors:** 


### ACADEMY OF MEDICINE AND SURGERY.

RICHMOND, VA., January 9th, 1894.

*Dr. Geo. Ben. Johnston, First Vice-President, in the chair.*

This being the first meeting of the new year the newly elected officers were regularly installed.

Dr. I. S. Welford, President.

Dr. W. S. Gordon, First Vice-President.

Dr. Virginius Harrison, Second Vice-President.

Dr. L. C. Bosher, Third Vice-President.

Dr. Jno. F. Woodward, Secretary and Reporter.

Dr. M. W. Peyser, Assistant Secretary.

Dr. J. M. Winfree, Treasurer.

Dr. Jas. N. Ellis, Librarian.

After the installation Dr. A. L. Welford read a paper on coughs.

He was followed by Dr. J. W. Long, who had been invited to exhibit a pathological specimen obtained from a *hysterectomy* that he had done a few days before. It was a case of interstitial fibroid, complicated with three months of pregnancy, occurring in a young negro woman. It is a rare operation, especially where the uterus, with its appendages, including the fetus in the unbroken membranes, is removed.

Malignant Pustule with Death Following a very Rapid Onset.

DR. A. L. WELFORD reported a case of anthrax without suppuration.

Mr. A., age twenty-six, came to his office January 7th, 1894, suffering intense pain and inflammation just over the malar region. There seems to have been a slight scratch at the seat of inflammation. The pain continued, accompanied by œdema and induration. Nothing seemed to afford relief. There were no signs of fluctuation or formation of pus. On Friday he made a crucial incision over the inflamed spot—the knife passing through a tough cartilaginous substance, with little or no discharge. A few heads of pus were seen superficially. The œdema increased, causing pressure upon the trachea; the pulse became weak, and a decided delirium set in. Sunday, pulse weaker, delirium increased, and by evening the patient was in collapse. Prior to this, however, the doctor called in Dr. Oppenheimer, and the incision was increased down to angle of the jaw, meeting with no collection of pus and very little hemorrhage. The patient was put on tonics in the beginning and every precaution used in operating, but to no avail. The man's health had not been good for several months. He had been a dealer in cattle, but not recently. The rapid fatality without formation of pus seems to make it a unique case.

DR. O. HARRISON reported the case of a lady who came to his office about four months ago with a chancre on the vulva. Her case was a sad one. Her husband having had syphilis, thinking himself cured, married. Three months after marriage the lady came to the doctor's office as seen above. He treated her from the primary stage—the secondary soon developed. When first seen she was about three months pregnant. Three weeks ago the doctor was called in and found she had albuminuria. Urine very scant, eight ounces in ninety-six hours. He prescribed various diuretics, with no effect. Two days later he gave a combination of calomel, squills and digitalis, with milk and lithia (Fonticello) water. In twenty-four hours she began to improve.

He wished to report the case for two reasons. First, to show the danger of advising marriage too soon after apparent cure of syphilis. Second, to open a discussion as to the advisability of inducing premature labor in a woman suffering from albuminuria to such an extent—suffocation from pressure of retained products upon the lungs—so that she found no comfort lying or sitting down. The doctor contemplated producing premature labor, but the symptoms abated so rapidly that he put it off. Ten days later, however, the patient aborted, the swelling having previously subsided. She made a rapid recovery. The dead fetus was macerated and showed in every vessel and organism syphilitic lesions.

DR. J. S. WELFORD thought the doctor would have been justified in bringing about premature labor under such circumstances.

